# Comparison of Friedewald, Martin/Hopkins, and Sampson formulae with direct LDL measurement in hyperlipidaemic and normolipidaemic adults in a Turkish population

**DOI:** 10.5937/jomb0-46549

**Published:** 2024-09-06

**Authors:** Medine Alpdemir, Mehmet Fatih Alpdemir, Mehmet Şeneş

**Affiliations:** 1 University of Health Sciences, Ankara Training and Research Hospital, Medical Biochemistry, Ankara, Türkiye; 2 University of Health Sciences, Ankara Bilkent City Hospital, Medical Biochemistry, Ankara, Türkiye

**Keywords:** low-density lipoprotein, Friedewald, Martin/ Hopkins, Sampson, niska gustina lipoproteina, Friedewald, Martin/Hopkins, Sampson

## Abstract

**Methods:**

The study was a retrospective investigation by the Department of Medical Biochemistry of the Ankara Training and Research Hospital between January 1, 2021, and December 31, 2022. Our study evaluated the results of 6297 patients aged 18-95 years who underwent cholesterol panel TC, TG, HDL-C, and direct LDL-C in our laboratory. The estimated LDL-C was calculated according to Friedewald, Martin/Hopkins, and Sampson formulae.

**Results:**

All three formulae showed a stronger positive correlation with d-LDL-C (0.905, 0.897, and 0.886, respectively, for all data, p<0.001). In addition, when we compared the total median difference (1st-3rd quartile) of all formulae, it was -0.69 (-1.62 to 0.39) for Friedewald, 0.034 (-0.74 to 1.14) for Martin/Hopkins and -0.40 (-1.19 to 0.55) for Sampson. According to Passing Bablok regression analyses, the intercept was determined as -0.97 (95% CI=-1.01 to -0.93), 0.41 (95%=0.37 to 0.44) and -0.05 (-0.08 to -0. 03) and slopes were calculated as 1.083 (95% CI=1.07-1.09), 0.88 (0.88 to 0.89) and 0. 90 (95%=0.89 to 0.90) for Friedewald, Martin/Hopkins and Sampson, respectively.

**Conclusions:**

Our findings suggest that the Martin/Hopkins formula performed better than the Friedewald and Sampson formulas. We figured out utilizing the Martin/ Hopkins formula as a good alternative for estimated LDLC in Turkish adults.

## Abstract (sr)

**Metode:**

Ova studija je bila retrospektivno istraživanje koje je sprovelo Odeljenje medicinske biohemije Bolnice u Ankari za obuku i istraživačke aktivnosti u periodu od 1. januara 2021. do 31. decembra 2022. Naše istraživanje je evaluiralo rezultate 6297 pacijenata uzrasta od 18 do 95 godina koji su prošli kroz panel holesterola TC, TG, HDLC, i direktnog LDL-C u našoj laboratoriji. Procenjeni LDL-C je izračunat prema Friedewald, Martin/Hopkins i Sampson formulama.

**Rezultati:**

Sve tri formule su pokazale jaču pozitivnu korelaciju sa d-LDL-C (0,905, 0,897 i 0,886, redom, za sve podatke, p<0,001). Takođe, kada smo uporedili ukupnu srednju razliku (od prvog do trećeg kvartila) svih formula, bila je -0,69 (-1,62 do 0,39) za Friedewald, 0,034 (-0,74 do 1,14) za Martin/Hopkins i -0,40 (-1,19 do 0,55) za Sampson. Prema analizama regresije Passing Bablok, presečna vrednost je određena kao -0,97 (95% CI=-1,01 do -0,93), 0,41 (95%=0,37 do 0,44) i -0,05 (-0,08 do -0,03), a nagibi su izračunati kao 1,083 (95% CI=1,07 do 1,09), 0,88 (0,88 do 0,89) i 0,90 (95%=0,89 do 0,90) za Friedewald, Martin/Hopkins i Sampson formule, redom.

**Zaključak:**

Naši nalazi sugerišu da Martin/Hopkins formula ima bolje performarse od Friedewald i Sampson formula. Ustanovili smo da je korišćenje Martin/Hopkins formule dobra alternativa za procenjeni LDL-C kod odraslih osoba u Turskoj.

## Introduction

The level of low-density lipoprotein cholesterol (LDL-C) is an important marker for the risk of coronary heart disease. High LDL-C levels increase the risk of developing coronary heart disease, while low LDL-C levels decrease the risk of coronary heart disease [1]. Routine measurement of LDL-C levels is recommended to determine the risk of coronary heart disease in both normolipidemic and hyperlipidemic individuals. In addition, LDL-C has long been an effective therapeutic target for the prevention of primary and secondary cardiovascular events [1] [2].

Both indirect estimation formulas (e.g., Friedewald, Martin/Hopkins, Sampson) and direct methods (homogeneous assay, electrophoresis, and sequential and density-gradient ultracentrifugation) are used to determine LDL-C levels. In all these formulas, LDL-C levels are estimated from triglyceride (TG), high-density lipoprotein cholesterol (HDL-C), and total cholesterol (TC) values (2). It is based on the Friedewald equation developed in 1972, which is commonly used to calculate LDL-C levels but has limited accuracy at low and very high triglyceride levels [3]. Formulas such as Martin/Hopkins and Sampson have been developed more recently to overcome the disadvantages of this formula [4] [5]. The Martin/ Hopkins formula uses an adjustable factor instead of a fixed TG denominator. This formula is more accurate than the Friedewald formula, especially at low LDL-C levels, and has shown a much stronger agreement with LDL-C measured directly by ultracentrifugation than the Friedewald formula in terms of TG level. However, the Martin/Hopkins LDL-C formula also has the disadvantage that it tends to overestimate the LDL-C level (or direct homogeneous LDL-C [d-LDL]), especially at high TG values [4] [5].

Recently, using the United States National Institutes of Health database, Sampson et al. derived a new LDL-C formula from 18,715 samples from 8656 patients [5]. They derived this formula using TG and non-HDL-C as independent variables with multiple least squares regression to calculate betaquantification and very-low-density lipoprotein cholesterol (VLDL-C) in a population with high TG. Compared to the Friedewald formula, the Sampson LDL formula provides more accurate results at higher triglyceride levels (<9.03 mmol/L).

The Friedewald, Martin/Hopkins, and Sampson formulas can be used to measure LDL-C levels in hyperlipidemic and normolipidemic adults. However, as the population-based performance of these formulas may vary, they need to be validated in different populations and compared with other laboratory techniques. In our study, we aimed to compare the d-LDL-C test with the Friedewald, Martin/Hopkins, and Sampson formulae in the Turkish population.

## Materials and methods

The study is a retrospective investigation of the Department of Medical Biochemistry of AnkaraTraining and Research Hospital. Our study evaluated the results of 6297 patients aged 18-95 years who underwent cholesterol panel TC, TG, HDL-C in our laboratory between January 1, 2021, and December 31, 2022 (58% female, 42% male). The study was approved by the University of Health Sciences Ankara Training and Research Hospital clinical research ethics committee (Acceptance date: 27/07/2022, No: 1007/2022) according to the principles of the Helsinki Declaration. The Laboratory Information Management System (LIS) obtained patient demographic and laboratory data. Pregnant women and patients with chronic diseases such as cancer and renal failure were excluded. Patients were divided into eight groups according to TG values (A: <1.13 mmol/L, B: 1.13–2.25 mmol/L, C: 2.26–3.38 mmol/L D: 3.39–4.50 mmol/L, E: 4.52–5.63 mmol/L, F: 5.65–6.76 mmol/L, G: 6.77–7.89 mmol/L, H: 7.90–9.02 mmol/L) and six groups according to the non-HDL values (A: <2.59 mmol/L, B: 2.59–3.34 mmol/L, C: 3.36–4.11 mmol/L, D: 4.14–4.89 mmol/L, E: 4.91–5.66 mmol/L, F ≥5.69 mmol/L).

### Biochemical measurements

Only patient data where blood samples were taken between 8.00 and 10.00 a.m. were used toexclude non-fasting persons as far as possible. Serum d-LDL-C levels were determined by the homogeneous direct measurement method (Roche Diagnostic, Indianapolis, IN, USA). TC, HDL-C, and TG levels were measured using a colorimetric enzymatic reaction (Roche Diagnostic, Indianapolis, IN, USA). The Centers for Disease Control LDL Cholesterol reference method laboratory network documented the traceability of the Roche Diagnostics GmbH CFAS lipids calibrators. Friedewald, Martin/Hopkins, and Sampson formulae were used for indirect LDL-C estimations (Table 1). Adjustable factor for Martin/ Hopkins was calculated based on TG and non-high-density lipoprotein cholesterol (non-HDL-C) levels derived from a 180-cell stratification table).

**Table 1:** Friedewald, Martin/Hopkins, and Sampson formulas.

	Formula
Friedewald (1)	TC – (HDL-C+ TG/2.2)
Martin/Hopkins (4)	TC – (HDL-C+ TG/adjustable factor)
Sampson (5)	TC/0.948-HDL-C/0.971-[TG/8.56+(TG*non-HDL-C)/2140-TG^2^]-9.44

### Statistical analysis

Variables were represented as N (%), mean (x̄) ± standard deviation (SD), or median (M) (25%–75% quartiles). Kolmogorov-Smirnov and Shapiro-Wilk tests were used to check the groups’ normality. Comparison between groups was performed with the Student’s T test or Mann-Whitney U test. The correlation of the methods was performed with the Spearman or Pearson correlation tests. The Bland–Atman approach assessed the differences between LDL-C equations and direct measurement, and the Passing-Bablok regression analysis was evaluated. A value of P <0.05 was considered statistically significant. SPSS IBM Statistics 26 (IBM SPSS, Chicago, USA) and Analyse-it (Analyse-it Software Ltd., Leeds, UK) were used for statistical analyses.

## Results

**Figure 1:**
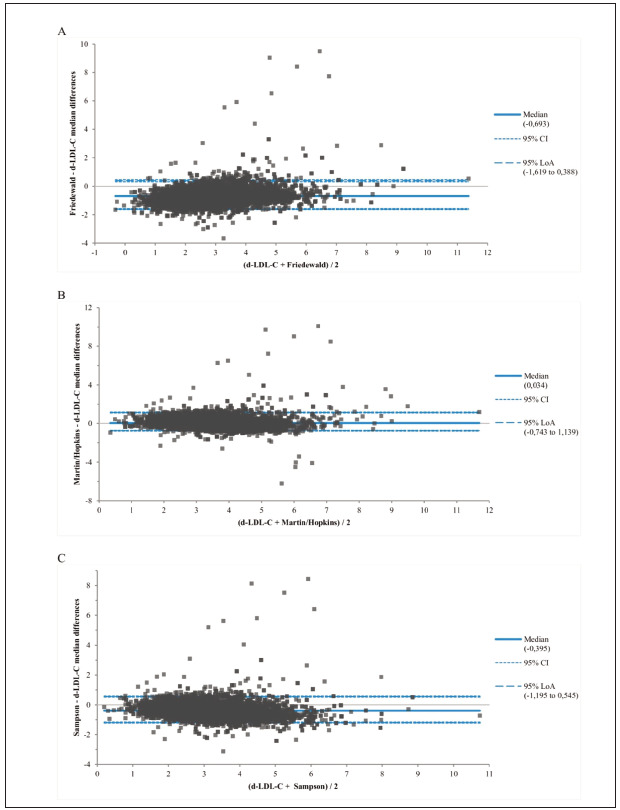
Differences between estimated LDL-C formulae and d-LDL-C results with Bland-Altman plots.<br>CI: Confident interval, LoA: limit of agreement of quartiles.

A total of 6297 patients’ [female: 3663 (%58.2) and male: 2634 (41.89)] results were included in thestudy. The age means and all the study variables data of all of the patients according to gender are shown in Table 2. Also, for each displayed variable to gender differences, there is a statistically significant difference (Table 2). Agreement between d-LDL-C and the formulae according to TG groups in the study population was assessed with the Bland-Altman test, and the median difference results are shown in Table 3. For the Friedewald formula, there was a negative median difference in all TG groups. The value of negative bias was found to increase in parallel with the increase in the concentration of TG. For the Martin/Hopkins formula, a negative median differenceat TG levels <4.52 mmol/L and a positive median difference were observed above this TG value. For the Sampson formula, there was a negative bias in the other TG groups except for the TG<1.13 mmol/L group. However, there was no increase in the negative bias dependent on TG concentration, and a constant bias was observed. In addition, when we compared the total median difference of all formulae, it was -0.69 (-1.62–0.39) for Friedewald, 0.034 (-0.74–1.14) for Martin/Hopkins, and -0.40 (-1.19–0.55) for Sampson for alt the patients (Figure 1).

**Table 2:** The age and baseline data of all patients and according to gender differences. This study presented variables as N (%), x̄±SD, or Median (1st–3rd quartile). d-LDL-C: measured low-density lipoprotein cholesterol, TC: Total Cholesterol, TG: Triglyceride, HDL-C: high-density lipoprotein cholesterol. Comparison between gender groups was used with the Mann-Whitney U test. The p-value shows the differences in the basic lipid parameters status by gender. P<0.05 was considered statistically significant.

	N: 6297	Female (N: 3663)	Male (N: 2634)	p-Value
Age (years)	52±13	54±13	50±13	
Friedewald (mmol/L)	2.59 (1.86–3.44)	2.74 (1.99–3.64)	2.42 (1.73–3.20)	<0.001
Martin/Hopkins (mmol/L)	3.32 (2.75–4.01)	3.41 (2.81 4.14)	3.22 (2.67–3.86)	<0.001
Sampson (mmol/L)	2.88 (2.29–3.60)	3.00 (2.37–3.77)	2.77 (2.18–3.40)	<0.001
d-LDL-C (mmol/L)	3.27 (2.59–4.06)	3.41(2.68–4.20)	3.11 (2.48–3.84)	<0.001
TC (mmol/L)	5.88 (5.15–6.75)	6.00 (5.22–6.90)	5.72 (4.99–6.54)	<0.001
TG (mmol/L)	5.06 (4.58–5.93)	5.01 (4.56–6.02)	5.10 (4.64–6.02)	0.284
HDL-C (mmol/L)	0.98 (0.83–1.14)	1.01 (0.88–1.22)	0.91 (0.78–1.06)	<0.001
Non-HDL-C (mmol/L)	4.86 (4.14–5.71)	4.94 (4.19–5.82)	4.78 (4.09–5.53)	<0.001

**Table 3:** Comparison of LDL formulae and d-LDL-C results according to TG groups. In this study, variables were presented as N (%), x̄ ± SD, or Median (1^st^–3^rd^ quartile). LoA: Limit of agreement. TG: Triglyceride, d-LDL-C: measured low-density lipoprotein cholesterol. Correlation between groups was used with the Spearman analysis (P<0.001). *P values indicate that a comparison between d-LDL-C and all formulae was used with the Mann-Whitney U test. P<0.05 was considered statistically significant.

Triglyceride, mmol/L	Median (1st–3rd quartile)	Median Difference<br>(Lower-Upper LoA)	r	p-Value*
Friedewald				
<1.13, n: 212	2.63 (2.08–3.25)	-0.286 (-0.85–0.15)	0.960	<0.001
1.13–2.25, n:403	3.29 (2.70–4.03)	-0.37 (-0.88–0.30)	0.948	<0.001
2.26–3.38, n:362	3.35 (2.75–4.17)	-0.63 (-1.11–(-0.10))	0.948	<0.001
3.39–4.50, n:306	2.89 (2.25–3.64)	-0.80 (-1.33–0.09)	0.943	<0.001
4.52–5.63, n:3037	2.62 (1.95–3.44)	-0.70 (-1.42–0.37)	0.909	<0.001
5.65–6.76, n:1269	2.24 (1.55–3.06)	-0.83 (-1.65–0.56)	0.867	<0.001
6.77–7.89, n:535	2.08 (1.32–2.91)	-0.85 (-1.93–0.66)	0.839	<0.001
7.90–9.02, n:173	1.67 (1.05–2.49)	-1.05 (-2.46–1.65)	0.764	<0.001
**Total**		**-0.69 (-1.62–0.39)**	**0.897**	**<0.001**
Martin/Hopkins				
<1.13	2.87 (2.22–3.52)	-0.208 (-0.82–1.00)	0.909	<0.001
1.13–2.25	3.61 (2.86–4.15)	-0.18 (-0.72–1.07)	0.896	<0.001
2.26–3.38	3.86 (3.21–4.52	-0.21 (-0.97–0.86)	0.864	<0.001
3.39–4.50	3.55 (2.99–4.29)	-0.18 (-0.88–1.00)	0.895	<0.001
4.52–5.63	3.39 (2.81–4.05)	0.08 (-0.72–1.01)	0.898	<0.001
5.65–6.76	3.15 (2.60–3.79)	0.12 (-0.75–1.26)	0.864	<0.001
6.77–7.89	3.12 (2.58–3.66)	0.14 (-0.69–1.32)	0.844	<0.001
7.90–9.02	2.92 (2.43–3.49)	0.22 (-1.08–2.62)	0.777	<0.001
**Overall**		**0.034 (-0.74–1.14)**	**0.886**	**<0.001**
Sampson				
<1.13	2.63 (2.08–3.30)	-0.27 (-0.77–0.21)	0.961	<0.001
1.13–2.25	3.39 (2.77–4.11)	-0.275 (-0.81–0.36)	0.949	<0.001
2.26–3.38	3.48 (2.91–4.26)	-0.502 (-39.20–1.75)	0.949	<0.001
3.39–4.50	3.10 (2.52–3.78)	-0.59 (-1.01–0.05)	0.943	<0.001
4.52–5.63	2.92 (2.35–3.62)	-0.39 (-1.13–0.50)	0.910	<0.001
5.65–6.76	2.65 (2.09–3.31)	-0.41(-1.36–0.69)	0.867	<0.001
6.77–7.89	2.58 (2.02–3.20)	-0.39(-1.38–0.70)	0.840	<0.001
7.90–9.02	2.33 (1.89–2.89)	-0.37 (-2.02–1.37)	0.764	<0.001
**Overall**		**-0.40 (-1.19–0.55)**	**0.905**	**<0.001**
d-LDL-C				
<1.13	2.90 (2.43–3.56)			
1.13–2.25	3.66 (3.00–4.40)			
2.26–3.38	3.98 (3.36–4.75)			
3.39–4.50	3.70 (3.00–4.43)			
4.52–5.63	3.33 (2.65–4.08)			
5.65–6.76	3.03 (2.39–3.81)			
6.77–7.89	2.89 (2.32–3.68)			
7.90–9.02	2.68 (2.13–3.56)			

The median difference was also evaluated according to non-HDL-C levels (Table 4). For the Friedewald formula, there was a constant negative bias in all groups. The median difference for the Martin/Hopkins formula was positive bias except for group 1. For the Sampson formula, except for group 1, the median difference was a negative bias in the other groups. The agreement between D-LDL and formulae for the total patient group was presented in the Bland-Altman plot (Figure 1). For both TG groups and non-HDL groups, the agreement between d-LDL-C and formulae was shown in Bland-Altman plots (supplemental Figure 1 for TG groups, supplemental Figure 2 for the non-HDL groups).

**Table 4:** Comparison of LDL formulae and d-LDL-C results according to non-HDL-C groups. This study presented variables as N (%) and Median (1st–3rd quartile). LoA: Limit of agreement, non-HDL-: non-high-density lipoprotein cholesterol, d-LDL-C: measured low-density lipoprotein cholesterol, correlation between groups was used with the Spearman analysis (P<0.001).*P values indicate that the Mann-Whitney U test compared d-LDL-C and all formulae. P<0.05 was considered statistically significant.

Non-HDL-C, mmol/L	Median<br>(1^st^–3^rd^ quartile)	Median difference<br>(Lower-Upper LoA)	r	p-Value
Friedewald				
<2.59. n: 126	1.63 (0.85–1.91)	-0.35 ( -2.05–(-0.03))	0.825	<0.001
2.59–3.34. n: 357	1.11 (0.67–2.32)	-0.66 (-1.64–0.08)	0.881	<0.001
3.36–4.11, n: 1045	1.61 (1.23–2.09)	-0.78 (-1.61–(-0.02)	0.825	<0.001
4.14–4.89, n: 1691	2.18 (1.84–2.55)	-0.75 (-1.68–0.003)	0.771	<0.001
4.91–5.66, n: 1445	2.85 (2.46–3.20)	-0.70 (-1.60–0.18)	0.757	<0.001
≥5.69, n: 1633	3.89 (3.43–4.55)	-0.55 (-1.54–0.99)	0.756	<0.001
Martin/Hopkins				
<2.59	1.69 (1.40–1.99)	-0.20 (-0.51–0.78)	0.912	<0.001
2.59–3.34	2.12 (1.81–2.56)	-0.004 ( -0.53–1.20)	0.841	<0.001
3.36–4.11	2.59 (2.27–2.92)	0.12 (-0.54–0.89)	0.807	<0.001
4.14–4.89	3.08 (2.78–3.38)	0.08 (-0.64–0.96)	0.743	<0.001
4.91–5.66	3.60 (3.25–3.93)	-0.01 (-0.76–1.12)	0.779	<0.001
≥5.69	4.40 (3.92–5.03)	-0.07 (-0.91–1.59)	0.755	<0.001
Sampson				
<2.59	1.66 (1.00–1.93)	-0.29 (-1.72–0.02)	0.837	<0.001
2.59–3.34	1.60 (1.28–2.36)	-0.31 (-0.88–0.25)	0.879	<0.001
3.36–4.11	2.06 (1.77–2.38)	-0.37 (-1.00–0.29)	0.829	<0.001
4.14–4.89	2.55 (2.30–2.85)	-0.38 (-1.11–0.35)	0.775	<0.001
4.91–5.66	3.12 (2.83–3.41)	-0.43 (-1.18–0.51)	0.759	<0.001
≥5.69	3.99 (3.61–4.57)	-0.45 (-1.40–0.98)	0.760	<0.001
d-LDL-C				
<2.59	1.88 (1.53–2.23)			
2.59–3.34	2.06 (1.60–2.61)			
3.36–4.11	2.49 (2.08–2.93)			
4.14–4.89	2.98 (2.58–3.45)			
4.91–5.66	3.56 (3.12–4.03)			
≥5.69	4.47 (3.84–5.09)			

**Figure 2:**
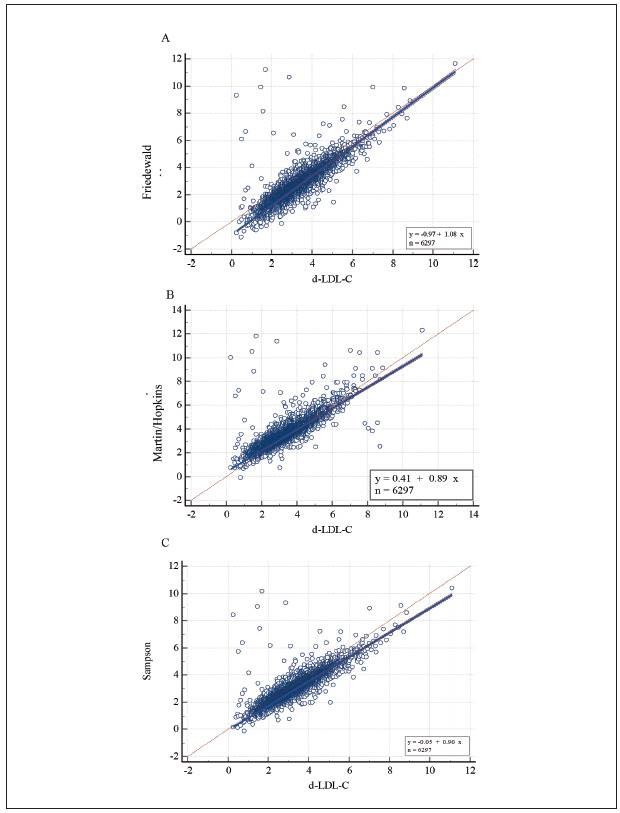
Passing Bablok regression analysis between estimated LDL-C formulae and d-LDL-C results for all the patients. A: Friedewald, B: Martin/Hopkins, C: Sampson.

There was a strong significant correlation between d-LDL-C and estimated LDL-C levels from formulae according to TG levels (p<0.001) (Table 3). Low TG levels (<4.52 mmol/L) had a relatively better correlation for all formulae (Table 3). The non-HDL-C groups observed a strong significant correlation between d-LDL-C and formulae (p<0.001). However, the correlation coefficient value was higher for all formulae at non-HDL values <4.14 mmol/L. A comparison of the correlation between d-LDL-C and formulae in non-HDL groups is presented in Table 4. The results of the correlation of formulae with d-LDL-C for all data sets are shown in Table 5. The Sampson formula had a better correlation than Friedewald and Martin/Hopkins formulae (0.905, 0.897, and 0.886, respectively, for all data, p<0.001).

**Table 5:** The results of the correlation among formulae with d-LDL-C for all data sets. d-LDL-C: measured low-density lipoprotein cholesterol. CI: confident interval. Correlation between groups was used with the Spearman analysis (p<0.001). P<0.05 was considered statistically significant.

	d-LDL-C<br>r (%95 CI)	Friedewald<br>r (%95 CI)	Martin/Hopkins<br>r (%95 CI)
Friedewald	0.897 (0.891–0.903)	-	-
Martin/Hopkins	0.886 (0.880–0.892)	0.903 (0.897–0.909)	-
Sampson	0.905 (0.899–0.911	0.995 (0.899–1.000)	0.915 (0.909–0.921)

According to Passing Bablok regression analyses, the intercept was determined as -0.97 (95% CI=-1.01 to -0.93), 0.41 (95%=0.37 to 0.44) and - 0.05 (-0.08 to -0. 03) and slopes were calculated as 1.083 (95% CI=1.07–1.09), 0.88 (0.88 to 0.89) and 0. 90 (95%=0.89 to 0.90) for Friedewald, Martin/Hopkins and Sampson for all the patients, respectively (Figure 2).

The rate of risk classification based on d-LDL-C measurement using a cut-off LDL-C >2.59 mmol/L, or minimal risk concentrations according to the recommendation of NCEP ATP III [6], was 75.3% based on d-LDL-C. The percentage agreement of the formulae was 49.9%, 81.4%, and 62.9% for Friedewald, Martin/Hopkins and Sampson, respectively.

## Discussion

Optimization of accurate estimation of LDL-C level is a critical and primary goal for CVD diagnosis and treatment. In this study, we compared the performance of measuring d-LDL-C with the recently developed Martin/Hopkins, Sampson, and traditional but still actively used Friedewald formulas. The present study found that the Sampson formula provided a relatively higher correlation, whereas the Martin/Hopkins formula showed a lower median difference. According to the Bland-Altman agreement, the Martin/Hopkins formula predicted slightly better LDL in both hyperlipidemic and normolipidemic subjects. In addition, there was a negative bias and underestimation in Friedewald’s all TG and Sampson formulas, whereas the Martin/Hopkins formula had an overestimated situation at TG>4.52 mmol/L. In our grouping by non-HDL-C, the Martin/Hopkins formula overestimated LDL-C, while the other formulas showed the opposite.

In our current study, our data suggest that the Martin/Hopkins formula shows better agreement with d-LDL-C results than Friedewald and Sampson according to the Bland-Altman test. Similar to our study, Azimi et al. [7] found that the Martin/Hopkins formula showed better agreement and lower bias when comparing these three formulas. In a systematic review and meta-analysis by Ephraim et al. [8], the Martin/Hopkins formula showed a better correlation value. A study by Rim et al. [9] indicated that compared to other formulas, Martin/Hopkins provided the best fit in a large Asian population cohort. Similarly, a study on the Korean population also demonstrated better results with Martin/Hopkins than other formulas; Friedewald, Hatta, Puavilai [10]. Although the Martin/Hopkins formula has proven to be a better assessment tool than other formulas, according to these studies and our study, it has several limitations. First, the impact of factors such as race/ethnicity, obesity, diabetes mellitus, and insulin resistance, which may affect the variance in the adjustable factor (TG/VLDL-C ratio), on the Martin/Hopkins formula has not been analyzed in all populations and situations. Secondly, it should be noted that there is a problem with standardizing the methods used in LDL-C measurement. The Martin/Hopkins formula must be validated using other LDL-C-quantification reference techniques in a larger population. Nevertheless, the Martin/Hopkins formula can be used as a remarkably accurate method for estimating LDL-C compared to the Friedewald formula.

The present study showed strong positive correlations with d-LDL-C for all formulae. The correlation intercept and slope are superior to Sampson than other formulae and in the majority of triglyceride and non-HDL subgroups and in the majority of triglyceride and non-HDL subgroups than other formulae. A study by Piani et al. [11] on a large, randomized, and blinded Italian population showed the Sampson equation provided a higher correlation with measured d-LDL-C level. Martínez-Morillo et al. [12], in Spain, suggested that the Sampson formula can be applied in clinical laboratories and provide acceptable performance. Few studies have compared the accuracy of Friedewald, Martin/Hopkins, and Sampson formulas in Turkish populations. Zararsız et al. [13] reported that the Martin/Hopkins approach is the method with the highest overall concordance according to LDL-C risk classifications. Again, in our previous study of the population of the Aegean region of our country, the Martin/Hopkins formula showed a better agreement with the direct method than the Friedewald formula. The population in this study includes the Central Anatolia region. Khan et al. [14] showed that the Teera kanchana formula had better correlation and agreement for the Pakistani population in a study comparing 13 formulae, including these formulas.

Many research studies have been conducted worldwide for these formulas for LDL-C calculation.However, no consensus has yet been reached on the most accurate and reliable formula for estimating LDL-C, especially for these two new formulas [15]. Direct measurement of LDL-C is costly and not commonly performed by clinical laboratories. The Friedewald formula is the most common method for LDL-C estimation. However, the Friedewald formula gives a lower estimate above TG levels of 3.39 mmol/L. Among the treatment targets set by the NCEP Adult Treatment Panel III, the lowest risk for LDL-C was recommended to be >2.59 mmol/L. The ACC/AHA guidelines recommend an LDL-C target of <1.81 mmol/L (<70 mg/dL) for atherosclerotic cardiovascular high-risk individuals [16]. In comparison, the European Society of Cardiology (ESC) and European Atherosclerosis Society (EAS) guidelines recommend more aggressive LDL-C targets [<1.42 mmol/L (<55 mg/dL)] [17]. According to NCEP ATP III, our evaluation’s percent agreement of the results was 75.3%, 49.9%, 81.4%, and 62.9% for d-LDL-C, Friedewald, Martin/Hopkins, and Sampson, respectively. The Martin/Hopkins formula appears least likely to misclassify patients due to the general overestimation of LDL-C values. Nevertheless, Friedewald and Sampson determined a higher misclassification and lower risk when evaluated according to d-LDL-C levels. In our study, Friedewald and Sampson’s formula had a negative bias. This suggests that, depending on the performance of the different formulae, less or more risk due to misclassification can lead to problems and making treatment decisions according to these.

Our research has some limitations. First, we utilized the direct homogeneous LDL-C measurement method instead of the gold standard beta quantification LDL-C method. However, the direct LDL-C method is used in most laboratories and has the analytical accuracy specified in the NCEP guidelines. Secondly, the data for this study were obtained from the laboratory results of a large-capacity public hospital in the capital of our country. However, although our number of patients is relatively large, it should be supported by validation studies with more individuals to generalize to the whole population due to ethnic or dietary differences between regions. Our last limitation is that although the data of our patients were screened with strict exclusion criteria with the developing technology, data on conditions such as race, ethnicity, obesity, fasting situations, and insulin resistance were not available in our system. These conditions may change the type of dyslipidemia in our patients. The strength of our study is that it is one of the rare studies conducted in our country. It is crucial to evaluate the performance of formulas in both normolipidemic and hyperlipidemic patient groups.

## Conclusion

In conclusion, we compared d-LDL-C measurement with formulas (Friedewald, Martin/Hopkins, and Sampson) in the Turkish population’s normolipidemic and hyperlipidemic individuals. The Martin/Hopkins formula showed an order of magnitude better LDL-C prediction. Although the Friedewald formula for LDL-C estimation is easy to remember, the Martin/Hopkins formula can easily estimate LDL-C without additional software, thanks to advances in laboratory information technology. To validate the Martin/Hopkins and Sampson formula in our population, both very large populations and additional studies considering factors such as race/ethnicity, obesity, diabetes, and insulin resistance are needed.

## Dodatak

### Author contributions

MA, MFA, MŞ design the study. MA collected the data. MFA analyzed the data. MFA wrote thedraft. MA, MŞ critically reviewed the manuscript.

### Financial support

None.

### Information on ethics committee approval

Our study protocol was approved by the Health Sciences University Ankara Training and Research Hospital Clinical Research Ethics Committee (Decision No: 1007/2022; 27/07/2022).

### Conflict of interest statement

All the authors declare that they have no conflict of interest in this work.
